# Evaluation of Inoculating Sterile Pericardial Fluid into Blood Culture Bottles

**DOI:** 10.1017/ash.2024.219

**Published:** 2024-09-16

**Authors:** Sky Cupid Douglas, Anne Matsko, Dana Pepe, Patrick Gordon, Matthew Lee

**Affiliations:** Beth Israel Deaconess Medical Center

## Abstract

**Background:** There is limited data regarding the benefits of direct inoculation of sterile pericardial fluid into blood culture bottles. We discovered widespread adoption of this practice at our institution during pericardiocenteses and became concerned about over-capturing of skin flora contaminants. We aimed to understand how organisms detected in pericardial fluid inoculated into blood culture bottles were interpreted clinically. **Methods:** We investigated a cluster of four patients with coagulase-negative Staphylococcus (CoNS) isolated in pericardial fluid inoculated blood culture bottles (PF-BCxBs) over a 2-week period; three of these patients had recent cardiac surgery and were initially flagged as potential SSIs. We further expanded to a retrospective review and identified 28 patients with ≥1 organism isolated from PF-BCxBs from 7/2021 to 6/2023. Clinical, microbiological, and pharmacy data were abstracted. The primary outcome was the proportion of patients with a clinically diagnosed infection. **Results:** Investigation into the initial cluster revealed a pseudo-outbreak - three of four patients had no clinical evidence of infection (CoNS was deemed a contaminant); one was treated for a potential infection. All patients had concomitant negative routine fluid cultures. Discussions with the cardiology teams revealed areas for improvement in the process for inoculating fluid into blood culture bottles. From the two-year review, 18% (5/28) of patients were clinically diagnosed with an infection (two Staphylococcus aureus; two CoNS; one Candida rugosa). Of the patients without Staphylococcus aureus, all three had a concomitant negative routine fluid culture, were receiving antibiotics prior to pericardiocentesis, and had white blood cell counts (WBC) >12 K/uL. The remaining 82% (23/28) of patients were deemed not to have an infection. Of these 23 patient without infection, organisms isolated were 16 CoNS (70%) and seven Cutibacterium species (30%). None of these patients had a fever, one (4%) was receiving pre-pericardiocentesis antibiotics, and three (9%) had WBC >12 K/uL. 70% (16/23) of these patients were started on antibiotics after gram-stain results; all were eventually discontinued (mean antibiotic days = 2, range 1-5 days). 83% (19/23) of these patients had a concomitant negative routine fluid culture. **Conclusion:** The majority of patients with an organism isolated from PF-BCxBs had either CoNS or Cutibacterium species and were deemed not to have a clinical infection. Within the small cohort limitations, clinical utility of blood culture bottle inoculation seems highest for patients with pre-procedural concern for infection. IPC teams should be aware of the non-pathogenic skin flora frequency and potential implication on SSI surveillance.

